# Effects of probiotic supplementation on post-infectious irritable bowel syndrome in rodent model

**DOI:** 10.1186/s12906-019-2610-9

**Published:** 2019-07-31

**Authors:** Ki-Bae Hong, Hyeyoung Seo, Joong-su Lee, Yooheon Park

**Affiliations:** 1 Department of Biological Sciences and Environmental Sciences Program, Southern Illinois University-Edwardsville, Edwardsville, IL 62026 USA; 2 Department of Integrated Biomedical and Life Science, Graduate School, Korea University, Seoul, 02841 Republic of Korea; 3 Department of Food Science and Biotechnology, Dongguk University, Goyang, 10326 Republic of Korea; 4 Probiotics center, Division of Healthcare, Daewon Pharmaceutical Company, 520 Cheonho-daero, Sungdong-gu, Seoul Republic of Korea

**Keywords:** Probiotics, Irritable bowel syndrome, *Trichinella*, Abdominal withdrawal reflex

## Abstract

**Background:**

Probiotics have been reported to be the active component used in the treatment of many functional gastrointestinal symptoms and syndromes. *Lactobacillus* and yeast culture are extensively used in probiotic supplements and traditional treatments for irritable bowel syndrome (IBS). The aim of this study was to investigate the effects of probiotic treatments (*Lactobacillus acidophilus* LA5, *Bifidobacterium animalis* subsp*. lactis* BB12 and *Saccharomyces cerevisiae* var*. boulardii*) on the behavioral response, targeted gene expression and pro-inflammatory cytokine levels of Pi (Post infectious)-IBS -induced mice.

**Methods:**

Pathogen-free male C57L/B6 mice and the *Trichinella*-infected mice were used to measure the score of abdominal withdrawal reflex (AWR). To compare molecular, biological and biochemical evidences of given probiotics with normal and positive control groups in mice, we conducted quantitative reverse transcription polymerase chain reaction (RT-qPCR), western blotting, and cytokine analysis.

**Results:**

Pi-IBS-induced immune response was confirmed that PAR-2 mRNA level was significantly increased by *Trichinella* infection (*P* < 0.05). The reduction of Pi-IBS symptoms through *Trichinella* infection and the effects of given probiotics were confirmed by a change in the protein levels of cytokines (*P* < 0.05). In addition, the administration of DW (Daewon) probiotics significantly decreased serum levels of IL-1 and IL-6 (*P* < 0.05).

**Conclusions:**

We have demonstrated that the given probiotics decreased pro-inflammatory cytokine levels in both the control and Pi-IBS induced mice. Taken all the results together, the results support that DW probiotics has a potential as a probiotic medication for patient with IBS via regulating TNF-α and IL-6 protein levels and serum IL-1 and IL-6 levels.

## Background

Irritable bowel syndrome (IBS) is a type of functional gastrointestinal disorder and a major public health problem that occurs in 9–23% of the population across the world [1, 2]. In addition, IBS is defined as common disturbances of the bowel characterized by abdominal pain or discomfort existing alongside abdominal distension. The four main types are categorized by the presence or absence of different symptoms such as constipation predominant (IBS-C), diarrhea predominant (IBS-D), mixed subtype (IBS-M) and unclassified (IBS-U) [3]. A meta-analysis has found that there exists a 3- to 11-fold higher risk of IBS after an episode of gastroenteritis [4]. There is increasing evidence that changes in the gastrointestinal (GI) microbiota are involved in the outbreak of IBS. Recent research suggests that food sensitivity, post-infectious reactivity, brain-gut interactions, alterations in GI tract and fecal microbiota, and upregulation of the GI mucosal immune system are related to a role for the GI microbiota in the pathogenesis of IBS [5, 6].

IBS therapies can be divided into medical treatments (antidiarrheals, antispasmodics, fiber supplements, and laxative agents) and modifications of the microbiota (probiotics and antibiotics) [7]. The current medical treatment strategy for IBS is based on symptom type and severity, and these treatments provide temporary symptom relief. Even though treatments have focused on over-the-counter medications to improve bowel habits, low costs, and safety, they have little impact on abdominal symptoms such as pain and bloating. Probiotics could alter the volume and/or composition of stool and gas or increase intestinal mucus secretion. These effects could influence intestinal handling of its contents and thus modulate symptoms such as constipation and diarrhea [8]. In clinical experience, the most efficacious utilization of probiotics in this field is the prevention of the post-infectious IBS (Pi-IBS) [9].

Pi-IBS is a common disease wherein IBS symptoms begin after acute gastroenteritis; this disease accounts for up to 4–30% of patients with bacterial gastroenteritis and seems to be a nonspecific response to infection [10]. Therefore, it is important to study the epidemiology and clinical features of Pi-IBS to understand the mechanisms that underlie it and to understand the treatments for this disorder. Pi-IBS is defined as the acute onset of new IBS symptoms in an individual who has not previously met the Rome criteria, immediately following an acute illness characterized by symptoms such as fever, vomiting, and alteration of stool form [11, 12]. In addition, Pi-IBS involved with *Salmonella*, *Campylobacter* and *E. coli* outbreaks, as well as bacteria and parasites are often used as Pi-IBS inducers in animal models. However, visceral hypersensitivity and alteration of motility and secretion, which are common characteristics of IBS, are weakly induced for Pi-IBS animal models infected by bacteria [13–15].

Experimental infection with the parasite, *Trichinella*, has been widely used to establish models for detecting the pathogenesis of intestinal dysfunction [16, 17]. A large number of studies have demonstrated that certain indicators, such as visceral hypersensitivity and the persistent dysfunction of the intestinal muscle, exist in mice infected with *Trichinella* spiralis [18]. Infection by *Trichinella* spiralis larvae induced changes in intestinal motility, visceral hyperalgesia, and intestinal mucus secretion [19]. These abnormal symptoms sustained after recovery from infection in a mouse model [20]. Accordingly, a *T. spiralis*-infected mouse is a suitable model of Pi-IBS.

In this study, *Trichinella* was used as a stimulant to induce Pi-IBS in a mouse model to evaluate the inflammatory response over the 4-week experiment to the intake of probiotic formulation. Thus, we have employed abdominal withdrawal reflex (AWR), quantitative reverse transcription polymerase chain reaction (RT-qPCR), western blot analysis, and cytokine analysis. The results suggest that probiotics formulated with two probiotic bacterial strains and yeast culture may be beneficial to human gastrointestinal health.

## Methods

### Animals

Four-week-old male C57L/B6 mice (15.5 ± 1.0 g) were obtained from Orient Bio Inc. (Gyeonggi-do, Republic of Korea). Animals were randomly divided into three groups, control group (control, *n* = 18), VSL3 group (VL, n = 18) and Daewon probiotic group (DW, n = 18). All animals were kept in a specific pathogen-free room at a temperature of 23 ± 2 °C in 50 ± 5% atmospheric humidity with 12-h light/dark cycles. The standard maintenance diet (Purina rodent chow) and tap water were freely provided in acrylic cages (Jeung do Bio & Plant Co., Ltd. Seoul. Korea) for 5 weeks and acclimatized for 1 week prior to use. Seven mice in each group were randomly selected for biological experiments and sacrificed by CO_2_ inhalation. All experiments were approved by the Ethical Committee and performed according to the guidelines and regulations of the Animal Care committee, Dongguk University (Seoul, Republic of Korea).

### Trichinella infection

Infective larvae were obtained from C57L/B6 mice infected with *Trichinella* at least 4 weeks in advance. The muscle containing encysted larvae were minced and digested in 1% pepsin A from porcine gastric mucosa (Sigma-Aldrich, St. Louis, MO, USA) and 1% HCl at 37 °C for 2 h. The isolated infective larvae were washed several times with PBS (Sigma-Aldrich). The Pi-IBS groups were infected by the oral administration of 250–300 larvae in 0.2 ml of solution, while the control group received the same volume of PBS.

### Probiotics treatment

Formulated probiotics (DW) were provided by Daewon Pharm Co. Ltd. (Seoul, Korea). VSL#3 (Farcoderma Srl, Italy) were purchased from a local pharmacy. Briefly, DW was composed of two strains, namely, *Lactobacillus acidophilus* LA5 and *Bifidobacterium animalis* subsp*. lactis* BB12 and yeast culture (*Saccharomyces cerevisiae* var*. boulardii*). VSL#3 was composed of eight probiotic bacterial strains, (*Streptococcus thermophilus*, *Bifidobacterium breve*, *Bifidobacterium longum* subsp. *longum*, *Bifidobacterium longum* subsp. *infantis*, *Lactobacillus acidophilus*, *Lactobacillus plantarum, Lactobacillus paracasei*, and *Lactobacillus delbrueckii* subsp. *bulgaricus*). After 2 weeks of inflammation, sample was diluted and spread on Petri dishes using BL and MRS agar to count and confirm the number of probiotics each mouse in acrylic cages was administered with probiotics (0.2 ml of the solution of 5.0 × 10^9^ CFU/g probiotics and yeast) and placebo solution, respectively, between 10:00 and 11:00 in a specific pathogen-free room. In addition, individual treatments were not revealed to the observers for oral administration [21].

### AWR (abdominal withdrawal reflex)

Mice were anesthetized with ether, and a distension balloon catheter (6-Fr, 2 mm external diameter) was placed in the descending colon of mildly sedated mice. After waking up and adapting for 20 min, each 10-s distention was followed by a 20-s resting period. Each level of distention (0, 0.35, 0.5 ml) was repeated two times, and the balloon was deflated and withdrawn after measuring AWR. The visceral sensory responses to rectal distension was quantified by scoring the AWR, as described in elsewhere [22].

### mRNA levels

RT-qPCR was used to measure epithelial-derived mRNA of protease-activated receptor-2 (PAR-2). Total RNA from intestinal tissues was isolated using the TRIzol® reagent (Invitrogen, CA, USA) according to the manufacture’s protocol. The concentration of total RNA was determined photometrically using a NanoQuant plate (Tecan Infinite 2000, Männedorf, Switzerland). Quality controlled RNA samples were treated with RQ1 RNase-free DNase I (Promega, WI, USA) and 1 μg of total RNA was reverse transcribed using SuperScript® III Reverse Transcriptase (Invitrogen). RT-qPCR was performed on the generated cDNA using the power Taqman PCR Master Mix kit (Applied Biosystems, CA, USA). Quantitative analysis was carried out using StepOne plus Software V. 2.0 (Applied Biosystems) and results were normalized to a validated control gene, GAPDH, using the ΔΔCt method [23].

### Western blotting analysis

Western blotting was performed to measure protein expression levels of cytokines by homogenizing intestinal cells in RIPA (Radio Immuno Precipitation Assay) buffer with protease and phosphatase inhibitor cocktail. Homogenized samples were then centrifuged at 10,000 rpm for 15 min. Protein concentrations in the supernatant of homogenates were measured using a Bradford assay. To denature the protein, loading buffer with the anionic denaturing detergent, sodium dodecyl sulfate (SDS), was used. After being diluted with loading buffer, the mixture was heated at 95 °C for 10 min. Depending on the molecular weights, 40 mg of protein lysate derived from intestinal samples were loaded onto 10% SDS-PAGE (Sodium Dodecyl Sulfate Polyacrylamide Gel electrophoresis) gel. Proteins were then transferred to nitrocellulose membranes and detected with a specific antibody against tumor necrosis factor-alpha (TNF-α; ab6671) and interleukin 6 (IL-6; ab208113) using the ChemiDoc™ imaging systems (Bio-Rad, Hercules, CA).

### Cytokine analysis

The blood serum samples were centrifuged, and then, the serum was separated and stored at − 80 °C. IL-1, IL-6 and TNF- α in serum were analyzed by quantification with MILLIPLEX® Multiplex assays according to the manufacturer’s instructions.

### Statistical analysis

Statistical analysis was determined using GraphPad Prism 5 (GraphPad Software Inc., La Jolla, CA, USA). All of the data are expressed as the mean ± standard deviation (SD). Differences between groups were evaluated by one-way analysis of variance (ANOVA) followed by Tukey’s multiple comparison test at a level of significance of *P* < 0.05. Single, double, and triple asterisk(s) were assigned to the experimental groups when they were significantly different from the control group at *P* < 0.05, *P* < 0.01 and *P* < 0.005, respectively.

## Results

### Effects on AWR score

The changes in AWR scores, according to *Trichinella* infection and probiotic administration during the 4-week period, are presented in Table 1. The AWR score for all experimental groups during the recording period showed an increase in volume dependence by CRD (0.25–0.5 ml water). After infection, 1-week PI groups showed a significant increase of AWR scores for distention (0.35–0.5 ml) (*P* < 0.05). In the comparison of 4-week PI control group, continuous probiotics administration significantly reduced both allodynia and hyperalgesia at 0.25–0.5 ml of CRD (*P* < 0.05). These results suggest that a decrease in visceral hypersensitivity was noted in two strains, *Lactobacillus* and yeast culture. Also, *Trichinella* infection and probiotic administration did not affect body weight, food intake, and drink volume (data not shown).

**Table 1:** Effect of given probiotics on abdominal withdrawal reflex (AWR) to colorectal distention (CRD)

Groups			N	Volume of colon distention		
				0.20 ml	0.30 ml	0.40 ml
1 weeks	Uninfected	Control	9	0.22 ± 0.34	1.18 ± 0.42	2.7 ± 0.33
		VL	9	0.23 ± 0.37	1.13 ± 0.31	2.4 ± 0.35
		DW	9	0.21 ± 0.33	1.09 ± 0.41	2.6 ± 0.39
	Infected	Control	9	0.53 ± 0.35	1.82 ± 0.34	3.3 ± 0.38
		VL	9	0.54 ± 0.33	1.84 ± 0.38	3.4 ± 0.24
		DW	9	0.48 ± 0.42	1.79 ± 0.39	3.3 ± 0.39
4 weeks	Uninfected	Control	9	0.22 ± 0.22	1.21 ± 0.27	2.5 ± 0.29
		VL	9	0.21 ± 0.33	1.19 ± 0.31	2.6 ± 0.26
		DW	9	0.21 ± 0.29	1.16 ± 0.28	2.4 ± 0.31
	Infected	Control	9	0.3 ± 0.42	2.29 ± 0.24	3.89 ± 0.22^a^
		VL	9	0.3 ± 0.30	1.71 ± 0.32	2.68 ± 0.40^b^
		DW	9	0.3 ± 0.34	1.52 ± 0.37	2.48 ± 0.29^b^

### Effects on PAR-2 transcription

**Fig. 1:**
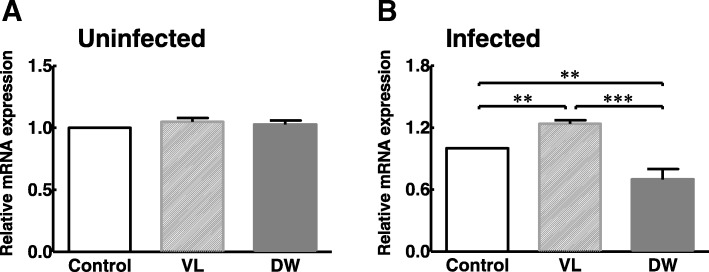
Intestinal mRNA levels of PAR-2 in Pi-IBS induced mice treated with or without probiotic treatments. VL: VSL3; DW: Daewon. Values are Mean ± SD (*n* = 9). Different symbols indicate significant difference at **p < 0.01 and ***p < 0.001

To examine whether the development of Pi-IBS through *Trichinella* infection and the effects of given probiotics caused a transcriptional process, the levels of mRNA for PAR-2 was determined (Fig. 1). Before *Trichinella* infection, the mRNA level of PAR-2 was not significantly decreased by both probiotic treatments and PAR-2 levels in infected animals were significantly increased in the control group when compared to the pre-infection control group (1.5 folds, *P* < 0.05) (Fig. 1a and  b). In the infected animals, transcript levels for the PAR-2 in the VL group were significantly higher than the control group (Fig. 1a, *P* < 0.01), but a probiotic treatment of DW significantly decreased PAR-2 mRNA level compared to control and VL groups (Fig. 1b, *P* < 0.01 and *P* < 0.001).

**Fig. 2:**
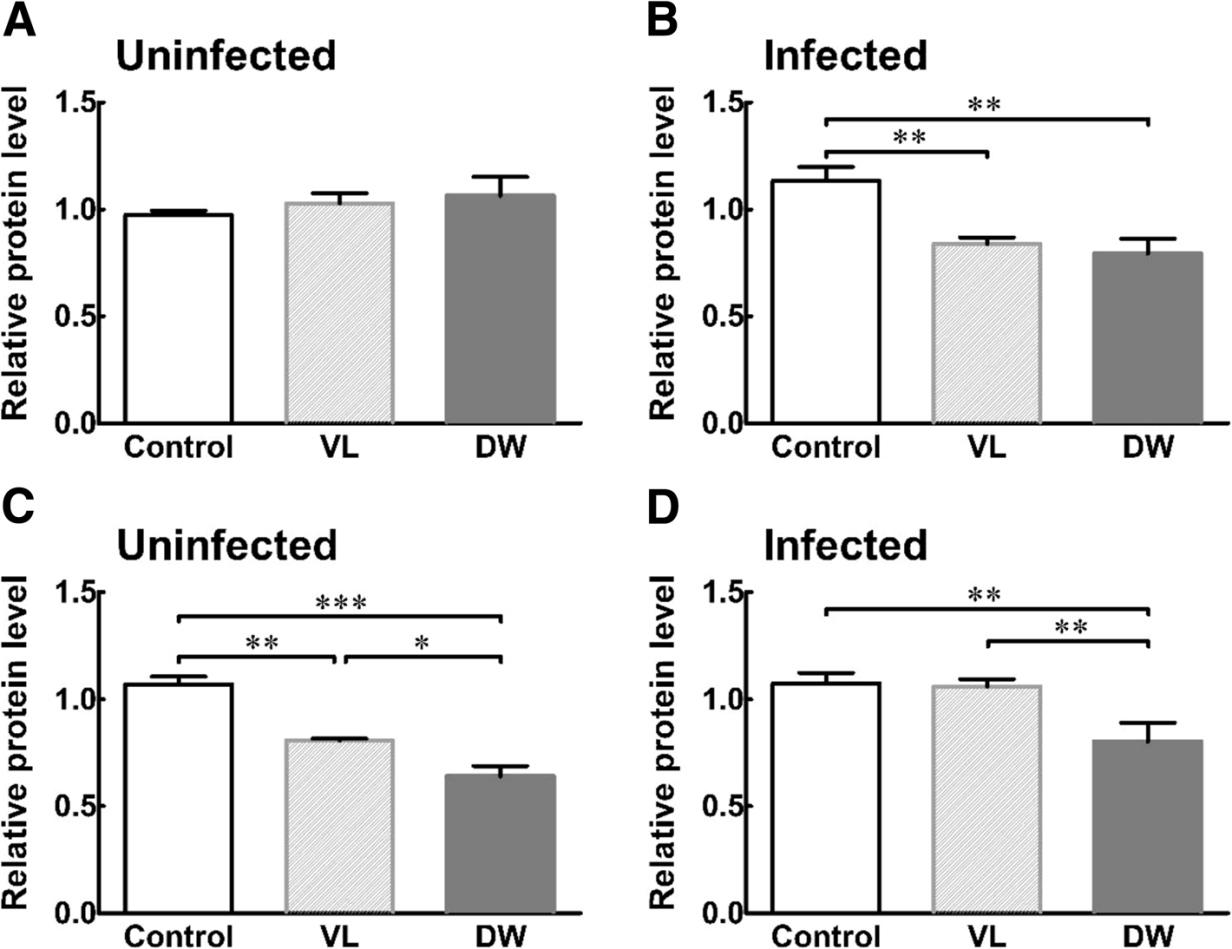
Intestinal TNF-휶 and IL-6 protein expression in Pi-IBS induced mice treated with or without probiotic treatments. VL: VSL3; DW: Daewon. Values are Mean ± SD (*n* = 9). Different symbols indicate significant difference at **p < 0.05, **p < 0.01 and ***p < 0.001

### Effects on TNF-α and IL-6 protein expression

The effects of DW treatment on intestinal TNF-α and IL-6 protein expression, induced by *Trichinella* infection, are shown in Fig. 2. The administration of all treatments had no effect on the levels of intestinal TNF-α compared to the control group. After *Trichinella* infection, the control group and VL group showed a significant increase in intestinal TNF-α levels, and these results showed that the increase in the pro-inflammatory cytokine probably resulted in enhanced acute mucosal inflammation by *Trichinella*. Intestinal TNF-α levels in the DW group was 1.34-fold lower compared to the control and VL groups (*P* < 0.05).

### Effects on serum cytokine levels

**Fig. 3:**
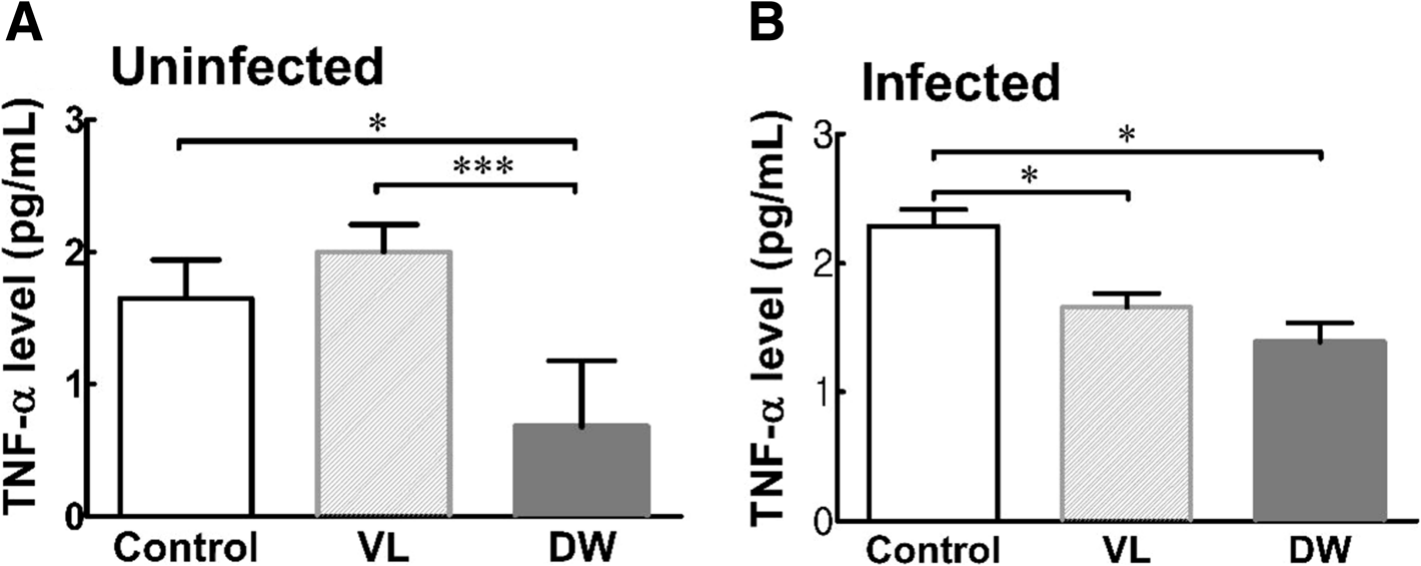
Serum level of TNA-α in Pi-IBS induced mice treated with or without probiotic treatments. VL: VSL3; DW: Daewon. Values are Mean ± SD (*n* = 9). Different symbols indicate significant difference at *p < 0.05 and ***p < 0.001

**Fig. 4:**
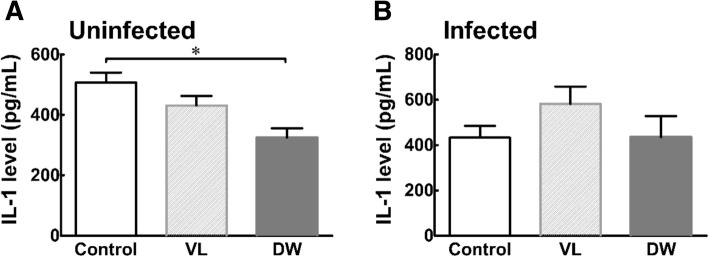
Serum level of IL-1 in Pi-IBS induced mice treated with or without probiotic treatments. VL: VSL3; DW: Daewon. Values are Mean ± SD (*n* = 9). Different symbols indicate significant difference at *p < 0.05 and ***p < 0.001

**Fig. 5:**
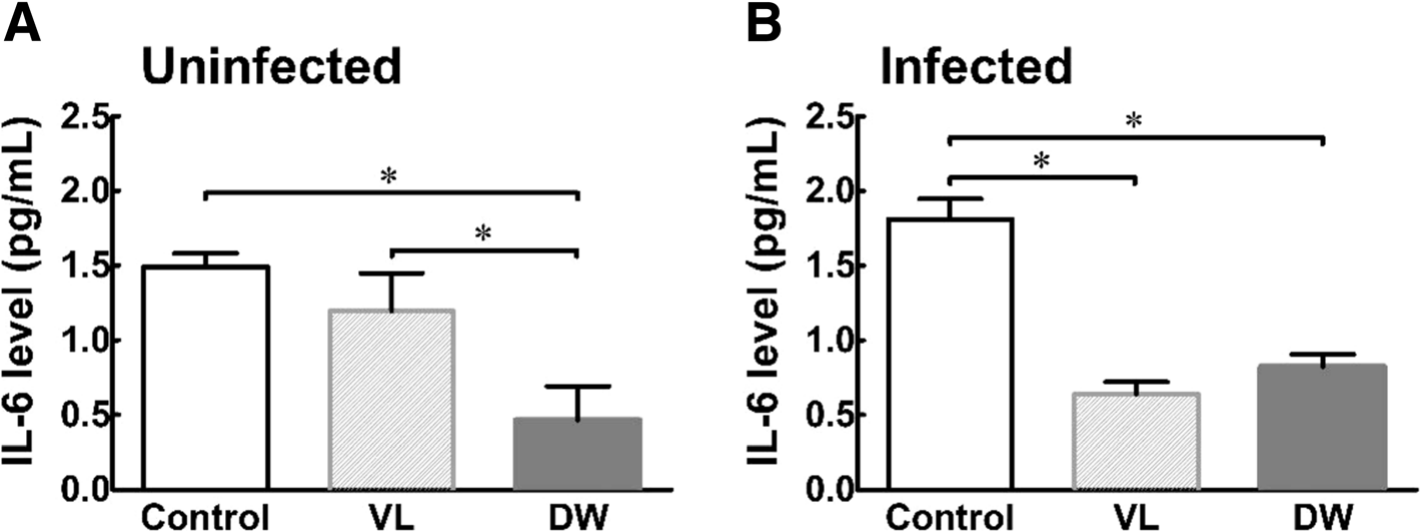
Serum level of IL-6 in Pi-IBS induced mice treated with or without probiotic treatments. VL: VSL3; DW: Daewon. Values are Mean ± SD (*n* = 9). Different symbols indicate significant difference at *p < 0.05 and ***p < 0.001

As shown in Figs. 3, 4 and 5, the levels of pro-inflammatory cytokines (TNF-α, IL-1, and IL-6) in the serum were measured. VL administration had no effect on the levels of TNF-α, IL-1, and IL-6 in the serum of uninfected group. DW administration caused significant reductions in TNF-α and IL-1 levels of uninfected mice compared to those in the control group (TNF-α and IL-1: *P* < 0.05) and VL group (TNF-α: *P* < 0.01). After *Trichinella* infection, VL group showed a significant increase in TNF-α, IL-1, and IL-6 levels in the serum (*P* < 0.05), and these results indicate that activated immune cells continue to release inflammatory cytokines after an infection. Although *Trichinella* infection to induce the Pi-IBS model, the serum cytokine levels of DW receiving mice were significantly reduced in comparison to the control and VL groups (*P* < 0.05).

## Discussion

Previous studies recognize that probiotics have health-beneficial effects on gut motility, immune modulation, pathogen exclusion, and epithelial barrier function. Additionally, probiotics are defined as live microorganisms which when administered in adequate amounts confer a health benefit on the host with gastrointestinal therapeutic potential, and they have been used in *Clostridium difficile* colitis, traveler’s diarrhea, antibiotic-associated diarrhea, inflammatory bowel disease, and IBS [21, 24]. Although IBS has a multifactorial etiology, and the exact mechanisms of how probiotics elicit the reductions of IBS symptoms are unknown, appropriate biomarkers are used for the evaluation of selected probiotic strains. The mechanisms of these health effects are also being revealed through in vitro and animal studies, which can be expected to lead to selection criteria for probiotics.

In the present study, we demonstrated the effects of given probiotics on colorectal distention through AWR assay (Table 1). Although approved live bacterial strains show great efficacy to gut microbiota, barrier function and the intracolonic short chain fatty acids (SCFAs), each strain is rarely used along for the treatment of IBS [25, 26]. Wang H et al. demonstrated that the groups of different probiotics and mixture (*Bifidobacterium longum*, *Lactobacillus acidophilus* and *Enterococcus faecalis*) markedly decreased AWR scores and contractile response, compared to PI-IBS mice [27]. Additionally, flatulence scores and retards colonic transit of patients with *Rome II* IBS were significantly reduced through the treatment of a composite probiotic (VSL# 3) [28].

Using RT-qPCR analysis, we studied the effects of given probiotics on target gene expression levels (Fig. 1). PARs belong to the family of G protein-coupled receptors (GPCRs) and are known to be associated with inflammatory conditions and tumor progression in previous studies. Additionally, PARs are expressed throughout the GI tract on several cell types, and activation of PAR-2 has been found on both the apical and basolateral side of colonic epithelial cells [29]. PAR-2 activation has been reported to cause the release of pro-inflammatory cytokines (e.g., IL-1β, IL-6, IL-8, TNF-α) and is important in the pathogenesis of organic and Functional GI disorders [30, 31]. In addition, Nébot-Vivinus M et al. demonstrated the relationship of probiotic dietary supplement (Lactibiane Tolerance®; consisting of *L. acidophilus*, *L. plantarum*, *L. salivarius*, and two stains of *B. lactis*) and epithelial barrier impairment with visceral hypersensitivity by IBS colonic fluid in C57BL/6 mice [32].

We also investigated the effects of given probiotics on the levels of pro-inflammatory cytokines, TNF- α and IL-6 (Fig. 2). Although cytokines are important signal transducers in the immune system, they regulate nerve function. Bertiaux-Vandaële N et al. announced that TNF-α causes marked sensitization to mechanical stimulation mediated by the interaction between TNFR1 and transient receptor potential A1 in IBS patients [33]. In addition, IL-6 is known to be a non-specific pro-inflammatory cytokine that influences adrenal stress response and gastric motility. Comparison studies of the genotypes and cytokines have shown that changes in IL-6 levels also related an acquired phenomenon and other potential gene polymorphisms [34].

Moreover, mice in probiotic treated groups showed significant regulations in serum levels of IL-1 and IL-6, compared to the mice in the control group (Figs. 3, 4, 5). Levels of IL-1β, TNF-α, and IL-6 have been found to be positively correlated with IBS symptoms in blood cytokine profiles in IBS patients. Wang IK et al. investigated the effect of probiotics administration on serum levels of cytokines and endotoxemia in peritoneal dialysis (PD) patients and the results showed that levels of serum TNF-α, IL-5, IL-6, and endotoxin significantly decreased after six months of treatment [35]. Additionally, *Lactobacillus rhamnosus* GR-1 and *Lactobacillus reuteri* significantly downregulated urinary TNF-α in the spinal cord injury patient [35]. In addition, levels of TNF-α, IL-1β, and IL-6 in peripheral blood mononuclear cells are correlated with symptom severity of IBS-D, including the intensity and frequency of pain and pain-related symptoms [36]. Shadnoush M et al. demonstrated that oral intake of 250 g of *Bifidobacterium* and *Lactobacillus* in the form of probiotic yogurt regulates the serum levels of IL-1β, IL-6, IL-10 and C-reactive protein (CRP) [37].

## Conclusion

In conclusion, we suggest that the Pi-IBS experimental approach can be used to assess the therapeutic potential of probiotic strains and assist IBS patients, based on the results. The effects of DW probiotics were associated with regulation of the protein levels of pro-inflammatory cytokines, TNF-휶 and IL-6, leading to the normalization of epithelial barrier integrity, and modulation of serum cytokine levels through a crosstalk between treatment and microbiota. The outcomes in this study are expected to provide behavioral, biochemical and molecular biological evidence for further understanding of probiotic strains and its impact on IBS symptoms.

## Data Availability

The dataset generated during the present study is available upon reasonable request to the author (Prof. Yooheon Park).

## Abbreviations

AWR: Abdominal withdrawal reflex
CRP: C-reactive protein
FGIE: Functional gastrointestinal disorder
GPCR: G protein-coupled receptors
IBS: Irritable bowel syndrome
IL: Interleukin
PAR-2: Protease-activated receptor-2
PD: Peritoneal dialysis
Pi-IBS: Post infectious irritable bowel syndrome
SCFA: Short chain fatty acid
TNF-α: Tumor necrosis factor-alpha
